# Association Between Residence in Historically Redlined Districts Indicative of Structural Racism and Racial and Ethnic Disparities in Breast Cancer Outcomes

**DOI:** 10.1001/jamanetworkopen.2022.20908

**Published:** 2022-07-08

**Authors:** Jesse J. Plascak, Kirsten Beyer, Xinyi Xu, Antoinette M. Stroup, Gabrielle Jacob, Adana A. M. Llanos

**Affiliations:** 1 Comprehensive Cancer Center, The Ohio State University, Columbus; 2 Division of Cancer Prevention and Control, Department of Internal Medicine, College of Medicine, The Ohio State University, Columbus; 3 Institute for Health and Society, Division of Epidemiology, Medical College of Wisconsin, Milwaukee; 4 Department of Statistics, The Ohio State University, Columbus; 5 Department of Biostatistics and Epidemiology, Rutgers School of Public Health, Piscataway, New Jersey; 6 Rutgers Cancer Institute of New Jersey, New Brunswick; 7 New Jersey State Cancer Registry, New Jersey Department of Health, Trenton; 8 Department of Epidemiology, Mailman School of Public Health, Columbia University, New York, New York; 9 Herbert Irving Comprehensive Cancer Center, Columbia University Irving Medical Center, New York, New York

## Abstract

**Importance:**

Historical structural racism may be associated with racial, ethnic, and geographic disparities in breast cancer outcomes, but few studies have investigated these potential relationships.

**Objective:**

To test associations among historical mortgage lending discrimination (using 1930s Home Owners’ Loan Corporation [HOLC] redlining data), race and ethnicity, tumor clinicopathologic features, and survival among women recently diagnosed with breast cancer.

**Design, Setting, and Participants:**

This cohort study used a population-based, state cancer registry to analyze breast tumor clinicopathology and breast cancer–specific death among women diagnosed from 2008 to 2017 and followed up through 2019. Participants included all primary, histologically confirmed, invasive breast cancer cases diagnosed among women aged at least 20 years and who resided in a HOLC-graded area of New Jersey. Those missing race and ethnicity data (n = 61) were excluded. Data were analyzed between June and December 2021.

**Exposures:**

HOLC risk grades of A (“best”), B (“still desirable”), C (“definitely declining”), and D (“hazardous” [ie, redlined area]).

**Main Outcomes and Measures:**

Late stage at diagnosis, high tumor grade, triple-negative subtype (lacking estrogen receptor, progesterone receptor, and human epidermal growth factor receptor 2 expression), breast cancer–specific death.

**Results:**

Among a total of 14 964 women with breast cancer, 2689 were Latina, 3506 were non-Latina Black, 7686 were non-Latina White, and 1083 were other races and ethnicities (non-Latina Asian/Pacific Islander/Native American/Alaska Native/Hawaiian or not otherwise specified); there were 1755 breast cancer–specific deaths. Median follow-up time was 5.3 years (95% CI, 5.2-5.3 years) and estimated 5-year breast cancer–specific survival was 88.0% (95% CI, 87.4%-88.6%). Estimated associations between HOLC grade and each breast cancer outcome varied by race and ethnicity; compared with residence in HOLC redlined areas, residence in HOLC areas graded “best” was associated with lower odds of late-stage diagnosis (odds ratio [OR], 0.34 [95% CI, 0.22-0.53]), lower odds of high tumor grade (OR, 0.72 [95% CI, 0.57-0.91]), lower odds of triple-negative subtype (OR, 0.67 [95% CI, 0.47-0.95]), and lower hazard of breast cancer–specific death (hazard ratio, 0.48 [95% CI, 0.35-0.65]), but only among non-Latina White women. There was no evidence supporting associations among non-Latina Black or Latina women.

**Conclusions and Relevance:**

Compared with redlined areas, current residence in non-redlined areas was associated with more favorable breast cancer outcomes, but only among non-Latina White women. Future studies should examine additional factors to inform how historical structural racism could be associated with beneficial cancer outcomes among privileged racial and ethnic groups.

## Key Points

**Question:**

Is historical mortgage lending discrimination (1930s redlining) associated with recent breast cancer outcomes differently by race and ethnicity?

**Findings:**

In this cohort study of 14 964 breast cancer cases, we found evidence that residence at diagnosis in areas historically graded “best” (vs residence in redlined areas) was associated with lower odds of late-stage diagnosis, lower odds of high tumor grade, lower odds of triple-negative subtype, and lower hazard of breast cancer–specific death, but only among non-Latina White women.

**Meaning:**

These findings suggest that historical structural racism may be associated with beneficial cancer outcomes among privileged racial and ethnic groups.

## Teaser

This cohort study examines the association of historical mortgage lending discrimination with racial and ethnic disparities in outcomes among women diagnosed with breast cancer from 2008 to 2017.

## Introduction

Historical and current structural racism are conceptualized as main drivers of cancer disparities by race and ethnicity,^1,2,3^ including poorer breast cancer outcomes experienced by US women self-identifying as Black or African American.^4^ Race-based policies and practices of exclusion such as Jim Crow laws in the South, housing covenants barring residents of color, and mortgage lending based on the percentage of residents from minoritized racial and ethnic groups in an area (ie, redlining) have contributed to poorer social and economic well-being among African Americans and minoritized racial and ethnic groups.^5,6,7,8,9^ Moreover, enduring place-based characteristics and policies (eg, locations of highways and other built environment factors; zoning codes; school district boundaries; municipal tax rates, revenues, and expenditures),^10,11,12,13,14^ as well as intergenerational transmission of socioeconomic factors, can indirectly translate decades-old structural racism into adverse health outcomes for people from minoritized racial and ethnic groups alive today who were not necessarily directly impacted by racist policies and practices that have since been abolished.^15,16,17^

There is a complex interplay among contemporary racial and ethnic stratification within residential areas,^18,19,20^ inequities in health care and built environment access,^21,22,23,24,25^ socioeconomic status,^26,27,28^ wealth,^26^ and health behaviors.^29,30,31^ These current factors may also be connected to historical processes involving mortgage lending discrimination.^7,31,32^ The now well-known 1930s Home Owners’ Loan Corporation (HOLC) mortgage security redlining maps of major metropolitan housing markets were originally created to assess lending risk across US cities and improve profitability of loans.^33,34,35^ Assessors assigned 1 of 4 grades: “best,” “still desirable,” “definitely declining,” or “hazardous” to neighborhoods partially based on the presence of “foreign-born” and “negro” residents.^35^ Analysis of the HOLC grading sheets indicates that historical presence of residents from minoritized racial and ethnic groups, poorer housing quality, average housing price, and older housing stock correlate with worse grading.^35^ Regardless of the debate over whether HOLC maps were subsequently used for mortgage lending decisions,^34,35^ there is general consensus that the racially prejudiced beliefs that motivated the HOLC maps also reinforced racial and ethnic stratification.

A small body of literature has begun to investigate associations between measures of mortgage lending discrimination by race and breast cancer outcomes.^36,37,38,39^ A study of breast cancer mortality within a Surveillance, Epidemiology, and End Results (SEER)-Medicare linkage found that greater likelihood of present-day redlining was associated with greater hazard of death among breast cancer cases.^38^ This association did not vary by race and ethnicity. Redlining values reflected mortgage applicants’ relative likelihood of loan denial within their residential census tract compared with denials outside their census tract.^40^ A study within the Metropolitan area of Atlanta, Georgia, found that greater redlining was associated with higher breast cancer–specific mortality among all women, with a potentially blunted association among non-Hispanic Black women.^37^ Another study investigated associations between HOLC, historical redlining, and breast cancer stage within areas of Massachusetts. Among 20 808 women diagnosed between 2001 and 2015, higher odds of late-stage disease was associated with residence in census tracts historically redlined compared with census tracts considered the lowest historical mortgage lending risk.^36^ This association was adjusted for race and ethnicity and most pronounced in census tracts characterized as economically and racially privileged (ie, high proportion of high-income and non-Latina White households), according to 2000 to 2017 census and American Community Survey data. A similarly framed Massachusetts-based study of incident breast cancer hormone receptor status found that the association between incidence of estrogen and progesterone negative tumors and residence in census tracts with historical redlining was dependent on current census tract economic and racial privilege^39^; albeit with highly mixed findings.

The racially directed nature of lending discrimination and evidence for racial and ethnic interactions from previous studies underscore the importance of analyses that test for variation in effect estimates by race and ethnicity. Moreover, to our knowledge, there is no literature reporting on associations between redlining and breast tumor grade or triple-negative breast cancer (TNBC)—both clinically relevant outcomes potentially influenced by social factors and which exhibit variation by race and ethnicity.^41,42^ Stress and inflammatory pathways have been proposed as possible biologic mediators between racism and health outcomes, including breast cancer outcomes, and potential contributors to racial and ethnic disparities.^1,43,44,45^ Accordingly, this study investigates associations between 1930s-era HOLC redlining, racial and ethnic identity, and breast cancer stage, grade, TNBC, and mortality among women diagnosed between 2008 and 2017 while a resident of a metropolitan area of New Jersey.

## Methods

### Study Design

This cohort study was conducted using a population-based cancer registry in compliance with the Strengthening the Reporting of Observational Studies in Epidemiology (STROBE) reporting guideline for cohort studies.^46^ The institutional review board of Rutgers University approved this study and informed consent was waived as data were from the state registry.

### Data Sources

Historical, georeferenced HOLC grading data were retrieved from the University of Richmond’s Digital Scholarship Lab.^47^ Assessments were dated between March 1937 and April 1940 and available within portions of 4 New Jersey counties (Bergen, Essex, Hudson, and Union) and 2 municipalities (Atlantic City and Trenton). Risk grades of A, B, C, and D corresponded to “best” (first grade), “still desirable” (second grade), “definitely declining” (third grade), and “hazardous” (fourth grade) within assessment sheets and color shading of green, blue, yellow, and red, respectively, in maps (Figure). An HOLC grade was attributed to each patient with breast cancer whose geocoded residential address at diagnosis fell within a historical HOLC area.

**Figure.**
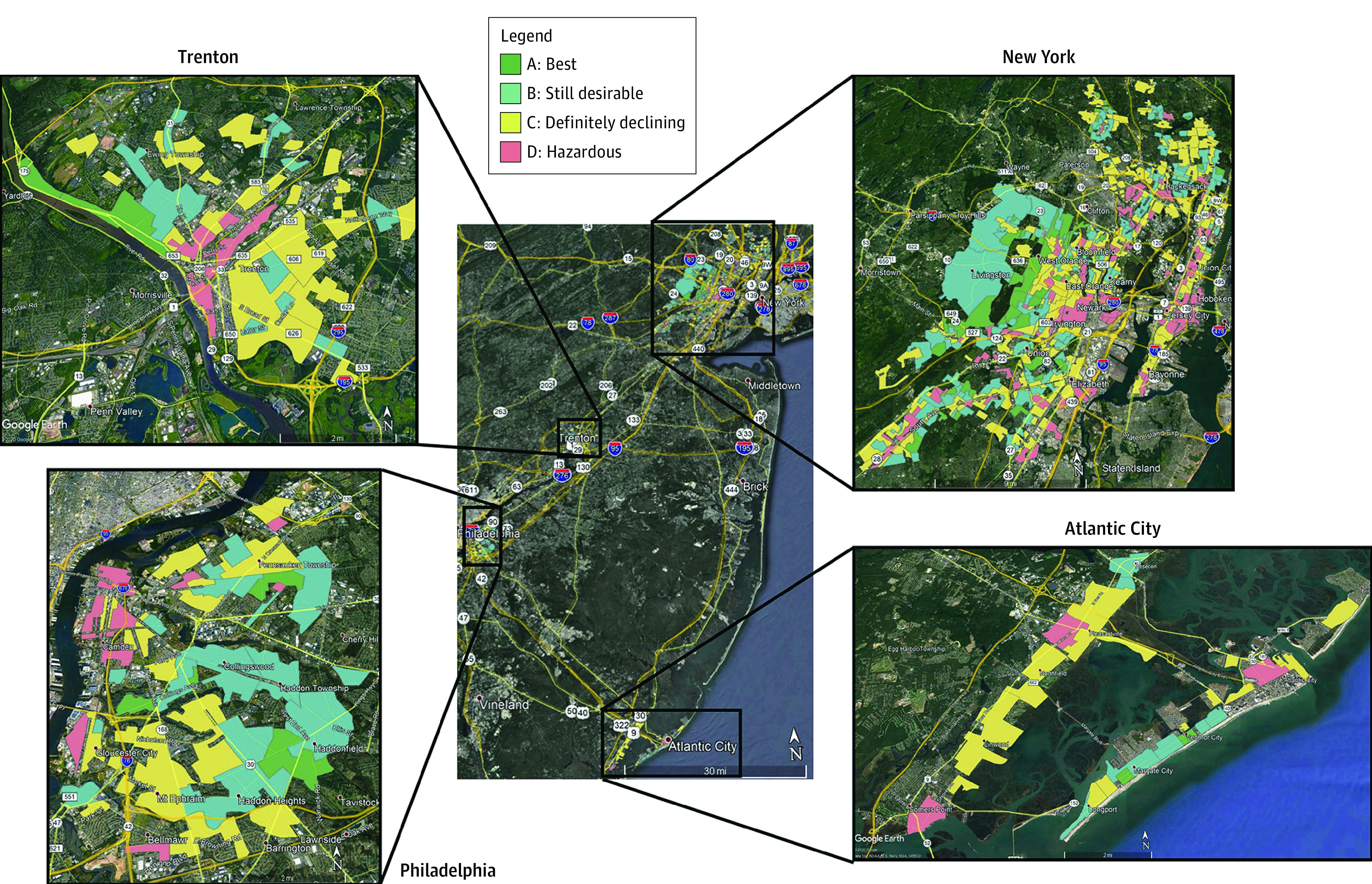
Historical Home Owner’s Loan Corporation Redlining Grades, 1937-1940, New Jersey

Cancer data were abstracted from the New Jersey State Cancer Registry (NJSCR) for all female residents of a HOLC-graded area, aged at least 20 years, diagnosed with a primary, histologically confirmed, invasive breast cancer between 2008 and 2017 (n = 15 025). Sociodemographic (age at diagnosis, race, ethnicity, geocoded residential address, date of diagnosis), tumor (stage at diagnosis, grade, subtype), and vital status (cause and date of death) data were also from the NJSCR. Age was collapsed into 5 categories: less than 40 years, 40 to 49 years, 50 to 64 years, 65 to 74 years, and at least 75 years. Race and ethnicity—primarily reported by health care facilities such as hospitals, physician offices, and other outpatient clinics like outpatient surgical centers (see eMethods in the Supplement for details)—were combined into Latina, non-Latina White, non-Latina Black, and other (non-Latina Asian/Pacific Islander/Native American/Alaska Native/Hawaiian and not otherwise specified). Stage at diagnosis was from SEER collaborative stage 2000, and any missing or unknown values were set to values from SEER summary stage 2000. Stage was dichotomized into early (localized and regional) and late (distant). Tumor grade was dichotomized into low (well and moderately well differentiated) and high (poorly differentiated and undifferentiated). Subtype was collapsed into TNBC (ie, negative for each of estrogen receptor, progesterone receptor), and human epidermal growth factor receptor 2) vs non-TNBC. Breast cancer–specific death was based on *International Statistical Classification of Diseases and Related Health Problems, Tenth Revision (ICD-10)* codes C50 to C50.9.

### Statistical Analysis

Race and ethnicity data were missing for 61 cases (0.4%), which were excluded from the analysis. Unknown and missing values for tumor subtype (1894 [12.7%]), grade (1620 [10.8%]), and stage (440 [2.9%]) were imputed using fully conditional specification multiple imputation resulting in 25 imputed data sets each with 14 964 cases.^48^ Cases missing follow-up time (151 [1.0%]) were included in multiple imputation analyses to maximize the number of non-survival outcomes but excluded from survival analyses. Sociodemographic and tumor factors were summarized by HOLC grades. Logistic regression models of tumor factors (TNBC, grade, stage) and Cox proportional hazard models of breast cancer–specific death were created, respectively, to investigate associations between outcomes, HOLC grade, and race and ethnicity while adjusting for age and year of diagnosis. Multiplicative interactions between HOLC grade and race and ethnicity were tested in each model of breast cancer outcomes. A random intercept for HOLC area was included in each model to account for clustered outcomes by HOLC area.^49^ Cases not experiencing breast cancer–specific mortality were right censored at the date of mortality from other causes or until end of follow-up, December 31, 2019. We calculated effect estimates (odds ratios [OR] from logistic models and hazard ratios [HR] from Cox models) and 95% CIs from each of the 3 tumor factor models and survival model. The proportional hazards assumption of Cox models was investigated through Schoenfeld residual plots, which indicated no violations. Multiple imputation results were combined and reported by the Rubin rule.^48^ As a sensitivity test of unstable estimates due to small frequencies, we collapsed grades A and B into 1 category and C and D into another category and repeated models. As post hoc analyses: (1) we calculated *F*-tests (logistic models) and χ^2^ tests (Cox model) to test whether estimated outcomes varied across HOLC grades, by racial and ethnic groups (see eResults in the Supplement), and (2) report observed and imputed sample size by HOLC grade and breast cancer outcomes (see eTable 1 and eTable 2 in the Supplement). We assessed statistical significance by magnitude of effect estimates (OR and HR) and statistical precision of effect estimates (95% CI). Analyses were conducted between June and December 2021 using ArcGIS version 10.6 (Esri) and SAS version 9.4 (SAS Institute).

## Results

Within each New Jersey metropolitan area with historical HOLC redlining grades available, worse HOLC grade areas were concentrated toward the urban cores as evident in Trenton, Camden, Atlantic City, and numerous populous cities in the Northeastern portion of the State (Newark, Elizabeth, Jersey City, Hoboken, Union City) (Figure).

There was a total of 14 964 women with breast cancer. Of these, 2689 were Latina, 3506 were non-Latina Black, 7686 were non-Latina White, and 1083 were other races and ethnicities (non-Latina Asian/Pacific Islander/Native American/Alaska Native/Hawaiian or not otherwise specified). Distributions of sociodemographic and tumor factors by residence within historical HOLC grades are shown in Table 1. Approximately 25% of non-Latina Black (26.1% [914 of 3506]) and Latina (27.7% [746 of 2689]) patients resided in “hazardous” HOLC grades compared with 12.5% of non-Latina White patients (964 of 7686); 69.4% of patients with late-stage diagnosis (761 of 1097) resided in the “definitely declining” or “hazardous” HOLC grades compared with 64.2% of patients with early stage (8615 of 13427) who resided in the “definitely declining” or “hazardous” HOLC grades; 67.9% of patients with high tumor grades (3547 of 5219) resided in the “definitely declining” or “hazardous” HOLC grades compared with 62.0% of cases with low tumor grades (5033 of 8125) who resided in the “definitely declining” or “hazardous” HOLC grades; 68.7% of cases with TNBC (1102 of 1603) resided in the “definitely declining” or “hazardous” HOLC grades compared with 63.3% of cases (7253 of 11467) without TNBC who resided in the “definitely declining” or “hazardous” HOLC grades.

**Table 1.** Distributions of Sociodemographic and Breast Tumor Factors by Historical HOLC Grade, New Jersey, 2008-2017 (N = 14 964)

Characteristic	Frequency and percentage of cases					
	Total		HOLC grade			
			“Best”	“Still desirable”	“Definitely declining”	“Hazardous”
Overall			1155 (7.7)	4131 (27.6)	6842 (45.7)	2836 (18.9)
Race and ethnicity						
Latina		2689 (18.0)	75 (2.8)	415 (15.4)	1453 (54.0)	746 (27.7)
Non-Latina						
Black	3506 (23.4)		217 (6.2)	768 (21.9)	1607 (45.8)	914 (26.1)
White		7686 (51.4)	809 (10.5)	2700 (35.1)	3213 (41.8)	964 (12.5)
Other^a^	1083 (7.2)		54 (5.0)	248 (22.9)	569 (52.5)	212 (19.6)
Age, y						
<40	899 (6.0)		51 (5.7)	200 (22.2)	427 (47.5)	221 (24.6)
40-49	2930 (19.6)		249 (8.5)	799 (27.3)	1351 (46.1)	531 (18.1)
50-64	5622 (37.6)		446 (7.9)	1564 (27.8)	2569 (45.7)	1043 (18.6)
65-74	2986 (20.0)		223 (7.5)	843 (28.2)	1348 (45.1)	572 (19.2)
≥75	2527 (16.9)		186 (7.4)	725 (28.7)	1147 (45.4)	469 (18.6)
Year of diagnosis						
2008-2011	5600 (37.4)		438 (7.8)	1519 (27.1)	2585 (46.2)	1058 (18.9)
2012-2014	4509 (30.1)		349 (7.7)	1303 (28.9)	2052 (45.5)	805 (17.9)
2015-2017	4855 (32.4)		368 (7.6)	1309 (27.0)	2205 (45.4)	973 (20.0)
Stage						
Early	13427 (89.7)		1070 (8.0)	3742 (27.9)	6137 (45.7)	2478 (18.5)
Late	1097 (7.3)		57 (5.2)	279 (25.4)	514 (46.9)	247 (22.5)
Missing	440 (2.9)		28 (6.4)	110 (25.0)	191 (43.4)	111 (25.2)
Grade						
Low	8125 (54.3)		715 (8.8)	2377 (29.3)	3547 (43.7)	1486 (18.3)
High	5219 (34.9)		334 (6.4)	1338 (25.6)	2522 (48.3)	1025 (19.6)
Missing	1620 (10.8)		106 (6.5)	416 (25.7)	773 (47.7)	325 (20.1)
Subtype						
Not triple-negative	11 467 (76.6)		946 (8.2)	3268 (28.5)	5213 (45.5)	2040 (17.8)
Triple-negative	1603 (10.7)		100 (6.2)	401 (25.0)	757 (47.2)	345 (21.5)
Missing	1894 (12.7)		109 (5.8)	462 (24.4)	872 (46.0)	451 (23.8)

Abbreviation: HOLC, Home Owners’ Loan Corporation.

^a^ Other race and ethnicity included Asian/Pacific Islander/Native American/Alaska Native/Hawaiian and not otherwise specified.

Median follow-up time was 5.3 years (95% CI, 5.2-5.3). With 1755 breast cancer–specific deaths the estimated 5-year breast cancer–specific survival was 88.0% (95% CI, 87.4%-88.6%). Estimated associations between HOLC grade and each outcome varied by race and ethnicity (Table 2). The race and ethnicity–dependent associations varied in a similar pattern across all 4 breast cancer outcomes; compared with residence in HOLC areas graded as “hazardous,” residence in historical HOLC areas graded “best” was associated with lower odds of late-stage diagnosis (odds ratio [OR], 0.34 [95% CI, 0.22-0.53]), lower odds of high tumor grade (OR, 0.72 [95% CI, 0.57-0.91]), lower odds of TNBC (OR, 0.67 [95% CI, 0.47-0.95]), and lower hazard of breast cancer–specific death (hazard ratio, 0.48 [95% CI, 0.35-0.65]) but only among non-Latina White women. Among non-Latina White women, residence in “still desirable” compared with “hazardous” or “definitely declining” compared with “hazardous” was also associated with lower odds of late-stage disease and lower hazard of breast cancer–specific death. The only other evidence of race and ethnicity–specific associations was among women from the other race and ethnicity category, which showed that compared with those residing in HOLC areas graded “hazardous,” residence in HOLC areas graded “still desirable” was associated with lower odds of late-stage diagnosis. Women with breast cancer who were at least 40 years of age had lower odds of late stage, high grade, and TNBC and lower hazard of breast cancer–specific death compared with those younger than 40 years (except for those at least 75 years of age in the late stage and survival models). Diagnosis in earlier years (2008 to 2011) was associated with higher odds of high grade and TNBC compared with more recent diagnosis. Results from models using HOLC grade collapsed into “best”/“still desirable” and “definitely declining”/“hazardous” were qualitatively similar. Results of post hoc analyses that formally test variation in estimated odds or hazard across HOLC grades by race and ethnicity support our results interpretation of Table 2 (eResults in the Supplement).

**Table 2.** Associations Between Late Stage, High Grade, TNBC, and Breast Cancer–Specific Death by Historical HOLC Grade and Sociodemographic Variables, New Jersey, 2008-2017

Factors	OR (95% CI)			Breast cancer specific-death, HR (95% CI)^d^
	Late stage^a^	High grade^b^	TNBC^c^	
HOLC “best” vs “hazardous”				
Non-Latina				
White	0.34 (0.22-0.53)	0.72 (0.57-0.91)	0.67 (0.47-0.95)	0.48 (0.35-0.65)
Black	1.02 (0.59-1.75)	1.01 (0.73-1.41)	1.06 (0.70-1.60)	0.78 (0.53-1.15)
Other^e^	0.81 (0.22-2.98)	0.84 (0.44-1.61)	0.78 (0.25-2.44)	1.07 (0.30-3.84)
Latina	0.72 (0.25-2.07)	0.71 (0.42-1.21)	0.49 (0.19-1.29)	0.88 (0.41-1.92)
HOLC “still desirable” vs “hazardous”				
Non-Latina				
White	0.65 (0.50-0.86)	0.94 (0.79-1.12)	0.82 (0.63-1.06)	0.60 (0.48-0.74)
Black	1.00 (0.69-1.43)	0.93 (0.74-1.16)	1.00 (0.76-1.30)	0.86 (0.67-1.10)
Other^e^	0.36 (0.14-0.96)	0.94 (0.63-1.42)	0.90 (0.46-1.76)	1.47 (0.70-3.10)
Latina	0.73 (0.43-1.24)	0.94 (0.72-1.21)	0.72 (0.48-1.08)	0.90 (0.61-1.33)
HOLC “definitely declining” vs “hazardous”				
Non-Latina				
White	0.72 (0.55-0.93)	1.15 (0.96-1.37)	0.93 (0.73-1.19)	0.74 (0.60-0.90)
Black	1.03 (0.75-1.40)	0.98 (0.80-1.19)	1.00 (0.79-1.27)	1.04 (0.85-1.27)
Other^e^	0.82 (0.43-1.59)	0.93 (0.65-1.32)	0.85 (0.47-1.52)	1.14 (0.58-2.26)
Latina	0.83 (0.57-1.20)	1.01 (0.83-1.23)	0.91 (0.69-1.20)	0.94 (0.71-1.25)
Age, y				
<40	1 [Reference]	1 [Reference]	1 [Reference]	1 [Reference]
40-49	0.56 (0.42-0.74)	0.58 (0.50-0.68)	0.75 (0.61-0.93)	0.61 (0.49-0.74)
50-64	0.83 (0.64-1.07)	0.53 (0.46-0.62)	0.70 (0.57-0.86)	0.72 (0.60-0.87)
65-74	0.73 (0.56-0.96)	0.38 (0.33-0.45)	0.61 (0.49-0.76)	0.77 (0.63-0.94)
≥75	1.11 (0.85-1.45)	0.37 (0.31-0.43)	0.56 (0.44-0.70)	1.42 (1.17-1.72)
Year of diagnosis				
2015-2017	1 [Reference]	1 [Reference]	1 [Reference]	1 [Reference]
2012-2014	0.92 (0.78-1.08)	1.20 (1.10-1.31)	1.06 (0.93-1.21)	0.92 (0.78-1.08)
2008-2011	0.97 (0.84-1.13)	1.23 (1.13-1.35)	1.26 (1.11-1.42)	0.97 (0.84-1.13)

Abbreviations: HR, hazard ratio; HOLC, Home Owners’ Loan Corporation; OR, odds ratio; TNBC, triple-negative breast cancer.

^a^ From logistic regression models of the probability of late stage (vs early) at diagnosis adjusted for all covariates listed, a random intercept for HOLC area, and accounting for multiple imputation variability.

^b^ From logistic regression models of the probability of high-grade tumor (vs low-grade) adjusted for all covariates listed, a random intercept for HOLC area, and accounting for multiple imputation variability.

^c^ From logistic regression models of the probability of triple-negative breast cancer (vs non-TNBC) adjusted for all covariates listed, a random intercept for HOLC area, and accounting for multiple imputation variability.

^d^ From a Cox proportional hazard model of breast cancer–specific death adjusted for all covariates listed and a random intercept for HOLC area, and accounting for multiple imputation variability.

^e^ Other race and ethnicity included Asian/Pacific Islander/Native American/Alaska Native/Hawaiian and not otherwise specified.

## Discussion

We tested whether current-day residence in historically demarcated areas that indicated mortgage lending discrimination by race and ethnicity (ie, redlining) was associated with recent breast cancer outcomes, and whether associations varied by race and ethnicity. Compared with residence in previously redlined areas, residence in non-redlined areas was associated with more favorable breast cancer outcomes—lower odds of late stage at diagnosis, high tumor grade, TNBC subtype, and lower hazards of breast cancer–specific death—almost exclusively among non-Latina White women. Most associations between redlining measures and breast cancer outcomes among breast cancer cases of other racial and ethnic groups had small effect estimates and wide confidence intervals, except for the lower odds of late stage comparing residence in areas deemed “still desirable” to redlined areas among women who were non-Latina Asian/Pacific Islander/Native American/Alaska Native/Hawaiian or not otherwise specified.

Other studies of HOLC redlining and breast cancer outcomes linked residential census tract at diagnosis to Massachusetts HOLC data and a Census Bureau–based measure of racialized economic segregation to investigate associations with late-stage and incident breast cancer by hormone receptor status.^36,39^ In the study of incident breast cancer by hormone receptor type, incidence of breast cancer was higher in non-redlined areas compared with redlined areas with the largest differences among census tracts considered to have the least present-day privilege—a result that was consistent across estrogen and progesterone receptor positive and negative tumors.^39^ In the study of late-stage breast cancer, there was evidence of an interaction involving racialized economic privilege such that proportion of late-stage diagnoses were lower in non-redlined areas compared with redlined areas most consistently among census tracts considered to have present-day socioeconomic privilege. A study of contemporary mortgage lending discrimination by race and ethnicity and breast cancer–specific death similarly found that residence in redlined areas was associated with a higher hazard of death only among non-Hispanic White patients with breast cancer.^37^

The few extant cancer epidemiology studies of residential redlining typically frame the role of racial discrimination as a health-adverse effect experienced by individuals identifying as a member of a minoritized racial or ethnic group, and who were targets of harmful practices and policies.^36,37^ Implied, but less discussed, is the potential for racial and ethnic disparities in cancer to persist due to historical efforts to preserve perceived benefits (ie, privilege) within neighborhoods that were overwhelmingly comprised of people identifying as White.^35^ This reorientation prompts consideration of how neighborhoods of mostly White people might have benefited in addition to or regardless of any harmful discriminatory effects experienced by individuals of a minoritized racial or ethnic group residing in redlined areas. This slight shift in perspective also allows for a common explanation of cancer inequities resulting from historical redlining, racialized housing covenants, and Jim Crow laws that have been legally abolished for decades—although with long-lasting impacts—as well as current, legal municipal zoning policies, public housing voucher allocations, and public school boundary delineations: each effectively maintaining perceived or actual advantages among already advantaged racial and ethnic groups.^10,11,12,13,14^

Thus, current-day residence in non-redlined areas might reflect historical access to better resources (eg, education, income, employment, wealth) that ultimately lead to more favorable breast cancer outcomes only among White populations. This might be due to intergenerational transmission of wealth and socioeconomic status not afforded to individuals residing in areas that were redlined or deemed less desirable for mortgage lending purposes. Relationships with additional factors that correlate with approximated HOLC boundaries, such as average levels of interpersonal discrimination should also be considered as previous research shows that residents who are among the numerical minority and self-identity as part of a minoritized racial or ethnic group report greater interpersonal discrimination as the proportion of their neighbors who identify as White increases.^50,51^ If this finding applied to the current study and non-redlined areas have higher proportions of White residents, then the breast cancer–beneficial mechanisms experienced by individuals from minoritized racial and ethnic groups residing in non-redlined areas could be offset by greater interpersonal discrimination ultimately leading to null associations between redlining and breast cancer outcomes for these residents who identify as part of minoritized racial and ethnic groups.

Potential biologic mechanisms underpinning relationships involving breast cancer outcomes, self-identified race and ethnicity, and interpersonal and structural racism could involve chronic psychosocial stress and genomic markers of inflammation (eg, DNA methylation or gene expression of interleukin receptors, C-reactive protein).^43,44,45^ Empirical studies designed explicitly to test whether stress and genomic inflammatory markers mediate associations between racism and breast cancer are lacking. However, one study has reported differential breast tumor methylation of numerous genes by neighborhood socioeconomic factors, with at least 1 such gene also correlating with mortality following a breast cancer diagnosis.^52^ Future studies of HOLC residence and cancer outcomes should strive to collect such additional measures as interpersonal discrimination, individual socioeconomic status, stress, and markers of inflammation to test these hypotheses.

### Study Limitations

In addition to unmeasured covariates for testing mechanistic pathways, this study is limited by the potential for HOLC exposure misclassification, geographic scope, and moderate sample size within specific strata. Although totaling nearly 15 000 total breast cancer cases, relatively few Latina women or women from other racial and ethnic groups resided in historical HOLC areas deemed “best,” which could have reduced the power to test associations. Factors specific to New Jersey—such as population migration and mobility, population density, racial and ethnic residential segregation—prevent generalization of these results to other areas. Exposure misclassification due to residential mobility patterns limit more complete characterization of the potential role of HOLC in breast cancer outcomes. Given the aforementioned potential mechanisms, it is expected that duration of residence would influence associations. Strengths include availability of data from a high-quality populous state cancer registry, linkage to the HOLC data set using geocoded residential addresses (as opposed to census tracts) to minimize geographic measurement error, and use of multilevel models and multiple imputation for improved accuracy of effect estimates.

## Conclusions

Historical redlining is one of many examples of structural racism that were ultimately motivated by the desire to maintain perceived advantages among people identifying as White. Although some portion of health inequities may be due to adverse effects experienced by targets of discrimination, racial and ethnic disparities in breast cancer prognostic factors and survival may also result from such practices and policies as historical redlining that disproportionately benefited White communities. This study highlights the importance of historical, race-based, conceptual framing of breast cancer disparities, and the importance of high-quality data that can be used to investigate such conceptualized relationships. Future studies should seek to replicate these findings in other US states and with estimates of exposure duration based on residential history data.

## Notes

Supplement. **eMethods.** Race and Ethnicity Data From the New Jersey State Cancer Registry **eResults.** Statistical Tests of Race-Ethnicity * HOLC Grade Interactions **eTable 1.** Observed Distributions of Breast Tumor Factors by Historical HOLC Grade and Race-Ethnicity, New Jersey, 2008-2017 (n=14,964) **eTable 2.** Imputed Distributions of Breast Tumor Factors by Historical HOLC Grade and Race-Ethnicity, New Jersey, 2008-2017 (n=374,100)
