# Supplementary material for: Association Between Residence in Historically Redlined Districts Indicative of Structural Racism and Racial and Ethnic Disparities in Breast Cancer Outcomes
Source: JAMA Netw Open. 2022 Jul 8;5(7):e2220908. doi: 10.1001/jamanetworkopen.2022.20908 (PMC9270695; doi:10.1001/jamanetworkopen.2022.20908)
Supplement: Supplement. — eMethods. Race and Ethnicity Data From the New Jersey State Cancer Registry eResults. Statistical Tests of Race-Ethnicity * HOLC Grade Interactions eTable 1. Observed Distributions of Breast Tumor Factors by Historical HOLC Grade and Race-Ethnicity, New Jersey, 2008-2017 (n=14,964) eTable 2. Imputed Distributions of Breast Tumor Factors by Historical HOLC Grade and Race-Ethnicity, New Jersey, 2008-2017 (n=374,100) [file jamanetwopen-e2220908-s001.pdf]

Supplemental Online Content

Plascak JJ, Beyer K, Xu X, Stroup AM, Jacob G, Llanos AAM. Association between residence in historically redlined districts indicative of structural racism and racial and ethnic disparities in breast cancer outcomes. *JAMA Netw Open.* 2022;5(7):e2220908. doi:10.1001/jamanetworkopen.2022.20908

eMethods. Race and Ethnicity Data From the New Jersey State Cancer Registry

eResults. Statistical Tests of Race-Ethnicity * HOLC Grade Interactions

eTable 1. Observed Distributions of Breast Tumor Factors by Historical HOLC Grade and Race-Ethnicity, New Jersey, 2008-2017 (n=14,964)

eTable 2. Imputed Distributions of Breast Tumor Factors by Historical HOLC Grade and Race-Ethnicity, New Jersey, 2008-2017 (n=374,100)

This supplemental material has been provided by the authors to give readers additional information about their work.

© 2022 Plascak JJ et al. *JAMA Network Open.*

eMethods. Race and ethnicity data from the New Jersey State Cancer Registry

The NJSCR requires that all reporting facilities submit information on race and ethnicity following North American Association of Central Cancer Registries standards. Race and ethnicity is primarily reported by healthcare facilities such as hospitals, physician offices, and other outpatient clinics like outpatient surgical centers. The NJSCR has no control over how these hospitals, physician offices, and other outpatient clinics record or report race-ethnicity, including whether race-ethnicity is strictly self-identified. To the extent that these reporting facilities collect (or fail to collect) race and ethnicity, the NJSCR may augment race and ethnicity data from other sources such as death certificates, National Death Index (NDI), and NJ Uniform Billing data (aka: hospital discharge data). NJSCR also augment race and ethnicity data from obituaries, electronic health records that are reported from physician offices and, if an individual is contacted for enrollment into a study, their race or ethnicity is updated if it is unknown in the registry. Currently NAACCR Gold certification standards require < 5% unknown race and NJSCR has been meeting that requirement. NJSCR is NAACCR Gold certified. For Hispanic and Asian/Pacific Islander groups, NJSCR uses the NAACCR Hispanic and Asian/Pacific Islander Identification Algorithm (NHAPIIA). This is a validated tool that improves Hispanic and API identification through the use of names, sex, birthplace information, and surname lists. These further increase the specificity of Hispanic and API classifications among cases that have unknown or non-specific Hispanic and API values.

© 2022 Plascak JJ et al. *JAMA Network Open.*

eResults. Statistical tests of race-ethnicity * HOLC grade interactions

P-values from F-tests (logistic models) or chi-square tests (Cox proportional hazard model) that test the null hypothesis that $\mu_{n1}=\mu_{n2}=\mu_{n3}=\mu_{n4}$, where μ is the odds or hazard, the first subscript 'n' is from 1 to 4 and denote the four racial-ethnic groups, and the second subscripts 1 to 4 denote HOLC grades levels 'Best', 'Still desirable', 'Definitely declining', and 'Hazardous' follow: we found evidence that odds of late-stage varies across HOLC grades among non-Latina White cases (P<0.001) and no evidence of variation among other racial-ethnic groups (all p > 0.20); odds of high grade varies across HOLC grades among non-Latina White cases (P<0.001) and no evidence of variation among other racial-ethnic groups (all p > 0.44); odds of TNBC varies across HOLC grades among non-Latina White cases (P = 0.03), weak evidence of potential variation across HOLC grades among Latina (p=0.12), and no evidence of variation across HOLC grades among other racial-ethnic groups (both p > 0.87); hazard of BrCa death varies across HOLC grades among non-Latina White cases (P<0.001) and no evidence of variation among other racial-ethnic groups (all p > 0.22).

© 2022 Plascak JJ et al. *JAMA Network Open.*

eTable 1. Observed distributions of breast tumor factors by historical HOLC grade and race-ethnicity, New Jersey, 2008-2017 (n=14,964)

	HOLC Grade, mean/n (sd/%)			
	'Best'	'Still desirable'	'Definitely	'Hazardous'
Among Non-Latina, White				
Stage				
Early	761 (10.9)	2463 (35.4)	2896 (41.7)	832 (12.0)
Late	29 (5.3)	180 (32.7)	243 (44.2)	98 (17.8)
Missing	19 (10.3)	57 (31.0)	74 (40.2)	34 (18.5)
Grade				
Low	534 (11.9)	1643 (36.6)	1753 (39.0)	560 (12.5)
High	196 (8.3)	789 (33.4)	1079 (45.7)	299 (12.7)
Missing	79 (9.5)	268 (32.2)	381 (45.7)	105 (12.6)
Subtype				
Not triple-negative	687 (11.1)	2203 (35.6)	2554 (41.3)	740 (12.0)
Triple-negative	54 (8.4)	215 (33.3)	284 (44.0)	92 (14.3)
Missing	68 (7.9)	282 (32.9)	375 (43.8)	132 (15.4)
Among Non-Latina, Black				
Stage				
Early	190 (6.2)	664 (21.8)	1396 (45.9)	793 (26.1)
Late	21 (6.4)	72 (21.8)	153 (46.4)	84 (25.5)
Missing	6 (4.5)	32 (24.1)	58 (43.6)	37 (27.8)
Grade				
Low	106 (6.4)	370 (22.5)	745 (45.3)	425 (25.8)
High	97 (6.7)	308 (21.3)	673 (46.4)	371 (25.6)
Missing	14 (3.4)	90 (21.9)	189 (46.0)	118 (28.7)
Subtype				
Not triple-negative	152 (6.3)	534 (22.2)	1121 (46.6)	599 (24.9)
Triple-negative	38 (6.6)	129 (22.3)	267 (46.2)	144 (24.9)
Missing	27 (5.2)	105 (20.1)	219 (42.0)	171 (32.8)
Among Non-Latina, A/PI/NA/AN/NH/Nos				
Stage				
Early	49 (5.0)	234 (23.7)	523 (53.0)	180 (18.3)
Late	3 (5.3)	6 (10.5)	34 (59.6)	14 (24.6)
Missing	2 (5.0)	8 (20.0)	12 (30.0)	18 (45.0)
Grade				
Low	31 (5.5)	133 (23.4)	298 (52.5)	106 (18.7)
High	19 (4.8)	90 (22.6)	211 (52.9)	79 (19.8)
Missing	4 (3.4)	25 (21.6)	60 (51.7)	27 (23.3)
Subtype				
Not triple-negative	47 (5.4)	202 (23.3)	464 (53.5)	155 (17.9)
Triple-negative	4 (5.1)	19 (24.1)	40 (50.6)	16 (20.3)
Missing	3 (2.2)	27 (19.9)	65 (47.8)	41 (30.1)
Among Latina				
Stage				
Early	70 (2.9)	381 (15.6)	1322 (54.0)	673 (27.5)
Late	4 (2.5)	21 (13.1)	84 (52.5)	51 (31.9)
Missing	1 (1.2)	13 (15.7)	47 (56.6)	22 (26.5)
Grade				
Low	44 (3.1)	231 (16.3)	751 (52.9)	395 (27.8)
High	22 (2.2)	151 (15.0)	559 (55.5)	276 (27.4)
Missing	9 (3.5)	33 (12.7)	143 (55.0)	75 (28.8)
Subtype				
Not triple-negative	60 (3.0)	329 (16.4)	1074 (53.5)	546 (27.2)
Triple-negative	4 (1.3)	38 (12.6)	166 (55.1)	93 (30.9)
Missing	11 (2.9)	48 (12.7)	213 (56.2)	107 (28.2)

© 2022 Plascak JJ et al. *JAMA Network Open.*

eTable 2. Imputed distributions of breast tumor factors by historical HOLC grade and race-ethnicity, New Jersey, 2008-2017 (n=374,100)

	HOLC Grade, mean/n (sd/%)			
	'Best'	'Still desirable'	'Definitely	'Hazardous'
Among Non-Latina, White				
Stage				
Early	19482 (10.9)	62886 (35.3)	74093 (41.6)	21570 (12.1)
Late	743 (5.3)	4614 (32.7)	6232 (44.1)	2530 (17.9)
Grade				
Low	14745 (11.7)	45519 (36.2)	49802 (39.6)	15698 (12.5)
High	5480 (8.3)	21981 (33.1)	30523 (46.0)	8402 (12.7)
Subtype				
Not triple-negative	18717 (10.8)	61459 (35.4)	72180 (41.5)	21381 (12.3)
Triple-negative	1508 (8.2)	6041 (32.8)	8145 (44.2)	2719 (14.8)
Among Non-Latina, Black				
Stage				
Early	4888 (6.2)	17319 (21.9)	36220 (45.8)	20651 (26.1)
Late	537 (6.3)	1881 (21.9)	3955 (46.1)	2199 (25.7)
Grade				
Low	2862 (6.2)	10504 (22.6)	21080 (45.3)	12070 (25.9)
High	2563 (6.2)	8696 (21.1)	19095 (46.4)	10780 (26.2)
Subtype				
Not triple-negative	4362 (6.2)	15536 (22.0)	32311 (45.8)	18347 (26.0)
Triple-negative	1063 (6.2)	3664 (21.4)	7864 (46.0)	4503 (26.3)
Among Non-Latina, A/PI/NA/AN/NH/Nos				
Stage				
Early	1272 (5.0)	6040 (23.6)	13364 (52.2)	4924 (19.2)
Late	78 (5.3)	160 (10.8)	861 (58.4)	376 (25.5)
Grade				
Low	831 (5.2)	3709 (23.4)	8288 (52.3)	3023 (19.1)
High	519 (4.6)	2491 (22.2)	5937 (52.9)	2277 (20.3)
Subtype				
Not triple-negative	1246 (5.0)	5695 (22.9)	13075 (52.7)	4799 (19.3)
Triple-negative	104 (4.6)	505 (22.3)	1150 (50.9)	501 (22.2)
Among Latina				
Stage				
Early	1773 (2.8)	9833 (15.6)	34152 (54.1)	17321 (27.5)
Late	102 (2.5)	542 (13.1)	2173 (52.4)	1329 (32.1)
Grade				
Low	1243 (3.2)	6251 (15.9)	20759 (53.0)	10943 (27.9)
High	632 (2.3)	4124 (14.7)	15566 (55.5)	7707 (27.5)
Subtype				
Not triple-negative	1745 (3.0)	9260 (15.9)	31370 (53.8)	15921 (27.3)
Triple-negative	130 (1.5)	1115 (12.5)	4955 (55.5)	2729 (30.6)

© 2022 Plascak JJ et al. *JAMA Network Open.*
